# The past, present, and future of myeloma staging and risk prognostication

**DOI:** 10.1093/oncolo/oyaf299

**Published:** 2025-09-19

**Authors:** Mehmet Baysal, Kelley Julian, Douglas Sborov, Amandeep Godara, Brian McClune, Jens G Lohr, Gliceida Galarza Fortuna, Ghulam Rehman Mohyuddin

**Affiliations:** Division of Hematology, Tekirdag Namık Kemal University, Tekirdag 59030, Türkiye; Division of Hematology, Huntsman Cancer Institute, University of Utah, Salt Lake City, UT 84112, United States; Division of Hematology, Huntsman Cancer Institute, University of Utah, Salt Lake City, UT 84112, United States; Division of Hematology, Huntsman Cancer Institute, University of Utah, Salt Lake City, UT 84112, United States; Division of Hematology, Huntsman Cancer Institute, University of Utah, Salt Lake City, UT 84112, United States; Division of Hematology, Huntsman Cancer Institute, University of Utah, Salt Lake City, UT 84112, United States; Division of Hematology, Huntsman Cancer Institute, University of Utah, Salt Lake City, UT 84112, United States; Division of Hematology, Huntsman Cancer Institute, University of Utah, Salt Lake City, UT 84112, United States

**Keywords:** multiple myeloma, staging, prognostication, R-ISS, R2-ISS, MASS, genomics

## Abstract

With the emergence of several new staging and risk stratification systems in myeloma, we have explored the evolution of these frameworks—past, present, and future. We examine how staging systems have evolved over time, the strengths of current models, and the limitations that future research can address to further improve prognostication.

## Graphical abstract

**Graphical Abstract:**
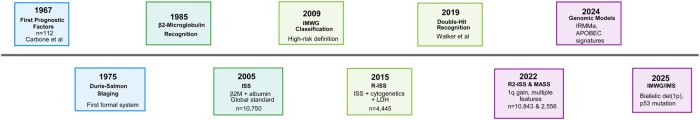

Implications for practice Contemporary myeloma staging systems provide better risk stratification than earlier models, enabling identification of high-risk patients for intensified therapy. However, current systems still have modest c-indices and fail to predict early progression in a minority of patients. While genomic models show promise, their clinical implementation faces cost and accessibility barriers. Improved staging remains essential for treatment selection, but limitations underscore the need for dynamic risk assessment and integration of post-treatment factors like measurable residual disease. Risk ascertainment in myeloma is complex and multi-faceted and while no single staging system can perfectly account for all of these factors, newer staging systems represent a significant step forward.

## Introduction

Although dramatic advances in therapeutic options have improved outcomes for patients with multiple myeloma, the disease is associated with considerable morbidity, and certain subsets of patients continue to experience poor outcomes.^1^ Staging for myeloma differs from that of solid tumors; regardless of myeloma stage, the mainstay of treatment remains systemic therapy, as opposed to local disease control through surgery or radiation. In myeloma, staging is more appropriately a form of risk stratification and prognostication, rather than true staging in the traditional sense. Specifically, myeloma staging helps us understand how responsive the disease may be to therapy, not necessarily the extent of disease present. In practice, patients with higher risk (higher stage) disease have been offered more intensive treatment for more prolonged periods of time, with the goal of remission and improving outcomes.^2–4^

**Figure 1.**
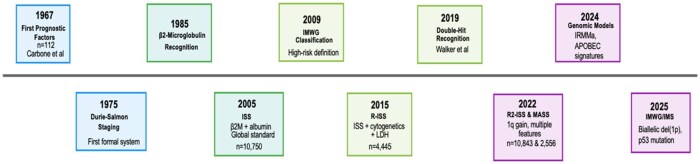
Chronological evolution of multiple myeloma staging systems.

In this review, we aim to summarize the past, present, and future of myeloma staging. We highlight how staging systems have evolved, what current staging systems do and do not capture, and potential criteria future staging systems may address. Figure 1 highlights the evolution of these staging systems.

## The past

### Early recognition of prognostic factors (1967-1975)

Myeloma has long been understood to be a heterogeneous disease, and historically, there have been many attempts to capture disease variations for prognostication and staging. As early as 1967, a retrospective study of 112 patients treated at a single center identified hemoglobin, serum calcium, and serum creatinine as predictive of outcomes.^5^ In 1969, Costa et al. proposed a staging system classifying patients as “good risk” (with respect to response to treatment with melphalan and prednisone and survival) if they met the following criteria: BUN < 30 mg/dL, serum calcium < 12 mg/dL, no evident sepsis, WBC > 4000 cells/μL, platelet count >100 000 per microliter of blood and an estimated survival of more than 2 months.^6^ A paper published in 1971 focused on renal function as a significant prognostic factor for outcomes.^7^ Other work in the early 1970s continued to identify markers of disease burden including anemia, hypercalcemia, renal failure, and high monoclonal protein levels whose presence was prognostic for poor outcomes. It was also recognized at this time that patients with a response to therapy as identified by a decrease in monoclonal protein had better outcomes than those who did not.^8^

### The Durie-Salmon era and its limitations (1975-2005)

The first formal staging system that was widely accepted was the Durie-Salmon staging, starting in 1975.^9^ This incorporated both laboratory markers and clinical markers, and stratified patients in 3 stages based on low, intermediate, and high cell mass, as demonstrated in Table 1.

**Table 1.** Durie-Salmon staging system.

Parameter	Stage I (low cell mass)	Stage II	Stage III (high cell mass)
	<0.6 × 10¹² cells/m²		>1.2 × 10¹² cells/m²
**Hemoglobin**	>10 g/dL	8.5-10 g/dL	<8.5 g/dL
**Calcium**	Normal or <10.5 mg/dL	—	>12 mg/dL
**M protein level**			
**Immunoglobulin G**	<5 g/dL	5-7 g/dL	>7 g/dL
**Immunoglobulin A**	<3 g/dL	3-5 g/dL	>5 g/dL
**Urine monoclonal protein**	<4 g/24 hours	4-12 g/24 hours	>12 g/24 hours
**Bone lesions**	Normal (scale 0)	—	Advanced lytic lesions (scale 3)
**Sub-classification**			
**A**	Serum creatinine <2.0 mg/dL		
**B**	Serum creatinine ≥2.0 mg/dL		

The Durie-Salmon staging system endured and became the dominant staging system for myeloma, remaining in clinical practice until it was supplanted by the international staging system (ISS) in 2005. It persevered despite having been derived from only 71 patients and the counting of lytic lesions on a skeletal survey being operator dependent, unfortunately making it prone to subjective variation.

Numerous attempts to improve and replace the Durie-Salmon staging system ensued. Other scoring systems included the Medical Research Council staging system published in 1980, which was derived from a dataset of 485 patients.^10^ It was simpler than the Durie-Salmon, relying only on 3 values for prognostication, the urea concentration in the blood, hemoglobin, and performance status, and stratified patients into 3 stages.^10^ Another scoring system published in 1980 was the Merline, Waldenstrom, and Jayakar staging system. This was derived from 201 patients, which incorporated serum creatinine, serum calcium, and bone marrow plasma cell percentage for those with non IgA myeloma; for those with IgA myeloma, the hemoglobin, serum calcium, and the M protein level were utilized.^11^

### The rise of beta-2 microglobulin, albumin, and the ISS (1985-2005)

As researchers continued to refine prognostication approaches, attention turned to specific biomarkers with stronger predictive potential. In the 1980s, the prognostic value of beta-2 ­microglobulin was increasingly recognized^12–14^; by 1985, the power for the Durie-Salmon staging system was being challenged, as it was shown be inferior to the prognostic ability of much simpler single lab values such as creatinine, hemoglobin, and beta-2 microglobulin.^15^ Another study in 1986 identified beta-2 microglobulin as superior to both the Medical Research Council staging and the Merline, Waldenstrom, and Jayakar staging systems. This study suggested that the prognostic value of beta-2 microglobulin and albumin together outperformed the Durie-Salmon staging system, thus setting the stage for future improvements.^16^

In 1990, a study of 612 patients enrolled on a Southwest Oncology Group trial confirmed that serum beta-2 ­microglobulin was the most robust prognostic factor for myeloma, and that the prognostic value could be improved further by combining with albumin and age.^17^

Recognizing this, the International Myeloma Working Group (IMWG) accumulated data from 10 750 patients across 17 countries and devised the ISS which combined 2 simple prognostic values, albumin and beta-2 microglobulin.^18^ The ISS is highlighted in Table 2. The most important advantages of ISS were that it consisted of 2 easily ascertainable lab parameters (albumin and beta-2 microglobulin) and led to a convenient and reproducible 3-stage classification.^18^

**Table 2.** ISS and R-ISS.

Stage	Criteria
**ISS**	
**I**	Serum β2M <3.5 mg/L and serum albumin ≥3.5 g/dL
**II**	Serum β2M <3.5 mg/L and serum albumin <3.5 g/dL OR serum β2M 3.5-5.5 mg/L, irrespective of serum albumin
**III**	Serum β2M >5.5 mg/L
**R-ISS**	
**I**	Serum β2M < 3.5 mg/L AND serum albumin ≥ 3.5 g/dL AND no high-risk chromosomal abnormalities by iFISH AND serum LDH < upper limit of normal
**II**	Not R-ISS stage I or III
**III**	Serum β2M ≥ 5.5 mg/L AND either high-risk chromosomal abnormalities [del(17p) and/or *t*(4; 14) and/or *t*(14; 16)] by iFISH OR serum LDH > upper limit of normal

Abbreviations: β2M, beta-2 microglobulin; iFISH, interphase fluorescence in situ hybridization; ISS, international staging system; LDH, lactate dehydrogenase; R-ISS, revised international staging system.

### Initial understanding of cytogenetics (1985-2014)

Since the publication of ISS in 2005, there was a tremendous gain in understanding of cytogenetic variability in myeloma and its impact on prognosis.^19^^,^^20^ As early as 1985, it was recognized that cytogenetic abnormalities observed on karyotype had prognostic value in myeloma.^21^ With the improvement in technology of fluorescence in situ hybridization (FISH), distinct cytogenetic abnormalities that were commonly found in myeloma were discovered, and their prognostic value realized.^22–24^ Since myeloma is incredibly diverse from a cytogenetic standpoint, these abnormalities conferred immense prognostic implications and therefore the IMWG proposed a molecular classification of myeloma in 2009. Standard-risk disease was classified as the absence of deletion (17p), translocation (4; 14) (p16; q32), or translocation (14; 16) (q32; q23), with high-risk being classified by the presence of at least one of these abnormalities.^25^ Additionally, lactate dehydrogenase had been identified as a prognostic marker as early as 1991.^26^ An analysis from Greece of 996 patients, showed that lactate dehydrogenase retained key prognostic discrimination capacity even amongst those receiving thalidomide,^27^ with a survival of 21 vs 51 months based on whether lactate dehydrogenase was high or low. Although the prognostic value of lactate dehydrogenase was well known by publication of the ISS in 2005, it was not included because on a multivariate analysis in the ISS dataset, it had less prognostic utility and identified smaller patient subsets relative to albumin and beta-2 microglobulin.^18^

In 2014, IMWG proposed a consensus for risk stratification in multiple myeloma by recommending incorporating both traditional ISS and cytogenetics in risk stratification.^28^ They acknowledged that other than the consideration of prolonged proteasome inhibitor based therapy in translocation (4; 14), there was no evidence to suggest altering therapy based on specific disease characteristics.

### The emergence of the R-ISS (2015 onward)

Recognizing that both lactate dehydrogenase and cytogenetic abnormalities could add to the prognostic value of ISS, in 2015, the revised ISS (R-ISS) was developed (Table 2).^29^ This was based on data from 4445 patients with newly diagnosed multiple myeloma who were enrolled in 11 international clinical trials from 2005 to 2012. This staging system successfully incorporated the previous molecular classification of myeloma. High-risk cytogenetic abnormalities defined in the R-ISS were translocations (4; 14) or (14; 16), or deletion of chromosome 17p.^29^

Limitations remained in the R-ISS. The main caveat of R-ISS is that it classifies the majority of patients into Stage II, and this group had incredible heterogeneity in outcomes.^30^ For example, to qualify for R-ISS Stage II, a patient could have no cytogenetic abnormalities or several, but all were considered in the same stage. Work in 2019 from Walker et al. would describe the additive adverse effect of having multiple high-risk cytogenetic abnormalities, now referred to as double or triple hit myeloma, setting the stage for a new generation of staging systems that would incorporate this crucial finding.^31^ Despite these shortcomings, the R-ISS remains widely used in practice and in current clinical trials.

As early as 2005, work from the University of Arkansas identified that an extra copy of chromosome 1q (1q gain) and resulting overexpression of CKS1B conferred a poor prognosis in myeloma.^32^ Further work from Fonseca et al. confirmed that this was present in a third of patients with myeloma and was associated with other high-risk features, such as translocation (4; 14).^33^ Although this was associated with poor prognosis, a multivariate analysis at this time did not show evidence for this to be an independent prognostic marker for myeloma. As the R-ISS did not include 1q gain, future work showing the key prognostic value of 1q gain would refine the validity of the R-ISS.^34^

The lack of uniformity in reporting and annotation of cytogenetic abnormalities made it challenging to standardize the assessment of 1q abnormalities across different studies and clinical settings.^35^ Additionally, there was considerable debate regarding the prognostic impact of 1q abnormalities with some studies suggesting it was not an independent prognostic factor when adjusted for other high-risk cytogenetic abnormalities.^36^ This inconsistency in findings contributed to the hesitancy in adopting +1q as a high-risk marker in earlier risk stratification systems.

Although FISH has been useful in detecting high-risk cytogenetic markers, it is limited by variability in plasma cell enrichment methods, probe selection, and cut-off values.^19^ Even within the United States, there is great variability in methodology and reporting cytogenetic abnormalities in myeloma.^37^ In addition, it is known that approximately 30% of patients with early relapse do not have any high-risk genetic abnormalities on FISH.^38^

The cut-off percentage of cells with a high-risk abnormality on FISH that truly implies high-risk status has been controversial. For example, it is evident that plasma cell populations have deletion 17p at varying proportions. It has been unclear how this diversity affects clinical results, despite its significance for prognosis. In 2007, the Intergroup Francophone du Myeloma described a 60% cut-off value (proportion of cells with the abnormality) for detecting 17p deletions.^39^ Later work from the University of Arkansas demonstrated that in those with an otherwise high-risk gene expression profile, a 20% cut-off value had prognostic value.^40^ In one of the largest studies on this topic, incorporating data from 1766 patients, a deletion 17p cancer clonal fraction of 55% was found to be the threshold above which poor prognostic outcomes were seen.^41^

### Gene expression profiling

Although not widely utilized or universally available, several gene expression profiles (GEPs) have been developed and analyzed. A 70-gene signature that divides patients into 2 risk categories and predicts outcomes was described by Shaughnessy et al. in 2007. This 70-gene classifier is known as GEP70 or UAMS70.^42^ In 2012, a new risk signature was developed to discriminate standard-risk versus high-risk patients, known as SKY92.^43^ SKY92 GEP has been tested in both the newly diagnosed and relapsed refractory setting and has also been clinically validated in several studies.^44–46^ SKY92 has been combined with R-ISS and provided further refinement when compared to each risk classification system separately.^47^ Despite continued refinement, SKY92 has yet to take off in routine clinical practice given limitations of present-day clinical availability and overall difficulty with application worldwide.

## Moving to the present

### Attempts to define high risk

Myeloma is a complex disease because multiple primary and secondary genetic events contribute to its progression. Although myeloma is considered a single disease, it represents different disease processes comprising of cytogenetically distinct abnormalities. Trisomies and IgH translocations are considered primary cytogenetic abnormalities. Secondary abnormalities including 1q gain, deletion 1p, deletion 13 and MYC translocations occur throughout the course of the disease.^35^^,^^48^^,^^49^ Myeloma also exhibits heterogeneity because the recurrence of identical molecular abnormalities is infrequent, and different subclones of the disease can evolve uniquely within the same patient.^50^^,^^51^ Although the R-ISS incorporated cytogenetic information, it only used 3 parameters (translocation 4; 14, 14; 16 and deletion 17p), and as such did not fully capture other variables.

Considering these developments and advancements in multiple myeloma treatment, the IMWG updated the definition of high-risk (HR) myeloma in 2016: translocations (4; 14), (14; 16) and (14; 20), deletion 17p, nonhyperdiploid, and 1q gain were identified as high-risk cytogenetic abnormalities.^52^ The consensus report also highlighted that in individuals with high-risk cytogenetic abnormalities, trisomies may reduce the negative effects of high-risk cytogenetic abnormalities, and better overall and progression-free survival could be linked to hyperdiploidy.^52^ Nevertheless, the exact impact of hyperdiploidy in the modern era is unclear, and in at least 1 key dataset, hyperdiploidy did not impact prognosis.^31^

### Recognition of multiple high-risk features and double-hit disease (2019 onward)

In 2019, a pivotal paper by Walker et al. enhanced our understanding of risk in myeloma. They identified that the risk imparted by multiple cytogenetic features was cumulative, with patients having multiple high-risk abnormalities (such as deletion 17p and translocation 4; 14) experiencing worse outcomes than those with just 1 cytogenetic abnormality.^31^ Another pivotal finding from Walker et al. was how 1q gain played a key additive effect in worsening prognosis when combined with other high-risk cytogenetic features.^31^ In the era of modern therapy, this category of “double-hit” myeloma with multiple high-risk cytogenetics continues to experience poor outcomes with therapy.^53^

The first staging system that aimed to incorporate the presence of multiple cytogenetic abnormalities was published in 2019 by the Intergroupe Francophone du Myélome, which included 6 key variables: deletion 17p, deletion 1p32, gain 1q, translocation (4; 14), trisomy 21, and trisomy 5, and assessed data from 1635 patients enrolled in 4 trials.^54^ Importantly, this was also the first scoring system to account for factors that imparted a “positive prognostic value” of trisomies in myeloma, allowing for trisomies to impart a lower risk of progression in this scoring system.^54^

In the meantime, key information regarding the prognostic value of 1q gain continued to accumulate, with initially conflicting data about whether 3 copies of 1q were sufficient to impart poor prognosis, or whether 4 or more copies (amp1q) were needed.^36^ Data from Emory’s large cohort of 1000 patients treated with triplet induction (bortezomib/lenalidomide/dexamethasone), autologous transplant and then maintenance, showed no adverse impact of 1q gain without concurrent high-risk cytogenetics, but did show an adverse impact of 4 or more copies, or when present in conjunction with other cytogenetic features.^55^ Conversely, a patient level meta-analysis of 2,596 patients from 3 trials (German-speaking Myeloma Multicenter Group [GMMG]: GMMG MM5 trial, EudraCT 2010-019173-16, and the Myeloma XI trial) showed an adverse prognostic impact of 1q gain, with no discernible difference whether there were 3 copies or 4 or more in terms of prognosis.^56^ Another meta-analysis of patients from the Myeloma IX and XI trials also showed that 1q gain was significantly associated with worse outcomes compared to normal 1q copy number status. Amplification of 1q was also linked to shorter PFS and OS compared to normal copy numbers of 1q, but there was no significant difference when compared to 1q gain.^57^

### Contemporary staging systems: R2-ISS and MASS (2022 onward)

Enough information has accumulated regarding both 1q gain and the presence of multiple cytogenetic abnormalities, therefore allowing the European Myeloma Network (EMN) to suggest R2-ISS (Second Revision of the International Staging approach) as a more effective and useful staging approach for myeloma in 2022.^58^ R2-ISS has 6 variables: ISS stage II, ISS stage III, lactate dehydrogenase, deletion 17p, translocation (4; 14), and 1q gain/amplification, and data from 10 843 patients with newly diagnosed multiple myeloma that were enrolled on clinical trials.

Almost concurrently, researchers at the Mayo Clinic established a novel staging approach for newly diagnosed Multiple Myeloma patients called the Mayo additive staging system (MASS).^59^ MASS incorporates high-risk immunoglobulin heavy chain locus (IgH) translocations, 1q gain/amplification, chromosome 17 abnormalities, ISS-III, and elevated lactate dehydrogenase. The MASS risk prognosis classification was produced from a retrospective analysis of 2556 patients from the Mayo Clinic from 2004 to 2019.^59^  Tables 3 and 4 highlight each of these staging systems in detail, whereas Table 5 highlights the key differences between them. Key areas of difference include the relative weightage of 1q gain, and the number of Stages (4 for R-2 ISS and 3 for MASS).

**Table 3.** Description of the MASS.

Risk feature	Definition	Score
**High-risk IgH translocations**	At least one of *t*(4; 14), *t*(14; 16), *t*(14; 20)	+1
**1q gain/amplification**	Gain or amplification of 1q	+1
**Deletion 17p**	Presence of deletion 17p	+1
**ISS stage III**	Beta-2 microglobulin >5.5 mg/L	+1
**Elevated LDH**	Elevated LDH	+1

MASS risk categories are as follows: Stage I: Total score 0, Stage II: Total score 1, Stage III: Total score ≥2.

Abbreviations: FISH, fluorescence in situ hybridization; IgH, immunoglobulin heavy chain; ISS, international staging system; LDH, lactate dehydrogenase; MASS, Mayo additive staging systems.

**Table 4.** R2-ISS score criteria.

Risk feature	Definition	Score
**ISS stage II**	Beta-2 microglobulin <3.5 mg/L and serum albumin <3.5 g/dL OR beta-2 microglobulin 3.5-5.5 mg/L, irrespective of serum albumin	+1.0
**ISS stage III**	Beta-2 microglobulin >5.5 mg/L	+1.5
**Deletion 17p**	Presence of deletion 17p	+1.0
**Elevated LDH**	Elevated LDH	+1.0
**Translocation t(4; 14)**	Presence of t(4; 14) on FISH	+1.0
**1q gain/amplification**	Presence of 1q gain/amplification on FISH	+0.5

R2-ISS risk categories are as follows: Stage I (low): Total score 0, Stage II (low-intermediate): Total score 0.5-1.0, Stage III (intermediate-high): Total score 1.5-2.5, Stage IV (high): Total score 3.0-5.0.

Abbreviations: FISH, fluorescence in situ hybridization; IgH, immunoglobulin heavy chain; ISS, international staging system; LDH, lactate dehydrogenase.

**Table 5.** Key differences between the R2-ISS and MASS staging systems.

Characteristic	R-ISS (2015)	R2-ISS (2022)	MASS (2022)
**Number of stages**	3	4	3
**Includes 1q21 gain/amplification**	No	Yes	Yes
**Points assigned to 1q abnormalities**	—	+0.5	+1.0
**Accounts for double/triple hit**	No	Yes	Yes
**High-risk IgH translocations included**	*t*(4; 14), *t*(14; 16)	*t*(4; 14) only	*t*(4; 14), *t*(14; 16), *t*(14; 20)

Abbreviations: IgH, immunoglobulin heavy chain; ISS, international staging system; R-ISS, revised international staging system; MASS, Mayo additive staging systems.

A recent real-world study comparing their performance in a US cohort showed similar performance of the 2 staging systems,^60^ and showed how they effectively recategorized the majority of patients that were previously R-ISS Stage II into more discrete staging systems. The reclassification of these R-ISS stage II patients now allows for more discrete higher or lower risk stages, leading to better prognostication.^60^

## Now and the future

### Current limitations and unmet clinical needs in 2025

Both MASS and R2-ISS represent a key incremental improvement over previous staging systems and are increasingly being utilized in practice today. A limitation since uncovered was that the deletion of chromosome 1p is not accounted for. It is increasingly being recognized that biallelic deletion of 1p confers particularly poor outcomes, with even a single allele abnormality having prognostic implications.^30^ The negative prognostic implications of deletion 1p may arise because of the deletion of specific tumor suppressor genes such as CDKN2C and FAM46C that are located in these regions.^61^

To address this and other recent advances in the genomic understanding of myeloma, the most recent International Myeloma Society risk stratification published in 2025 classifies high-risk myeloma as having a deletion of chromosome 17p or TP53 mutation, as well as biallelic deletion of chromosome 1p.^62^ Additionally, the presence of any 2 of the following factors can categorize a patient as high-risk: chromosomal gain or amplification of chromosome 1q, monoallelic deletion of chromosome 1p, or IgH translocations, specifically translocations (4; 14),(14; 16) and (14; 20), beta-2 microglobulin greater than 5.5 mg/L, coupled with normal renal function.^62^

Despite many improved attempts at staging in myeloma, previous iterations historically have primarily served as a risk prognostication system, helping to predict outcomes rather than dictating specific treatment strategies.^63^ Recent trials specifically enrolling high-risk patients are now allowing novel combinations for patients with high-risk disease such as the 4 or 5 drug combinations explored in GMMG-CONCEPT^64^ and OPTIMUM/MUKnine.^65^

The absolute predictive values of modern staging systems remain suboptimal. A perfect staging system (albeit impossible) would theoretically have a c-index of 1.0 and inherently predict outcome, whereas a c-index of 0.5 is no better than chance. In practice, a c-index of at least 0.7 is considered the hallmark of a good staging system.^66^ As noted in a real-world cohort, both MASS and R2-ISS had a c-index of 0.6, which remains subpar for prognostication and highlights the need for more robust staging systems in the future.^60^

**Figure 2.**
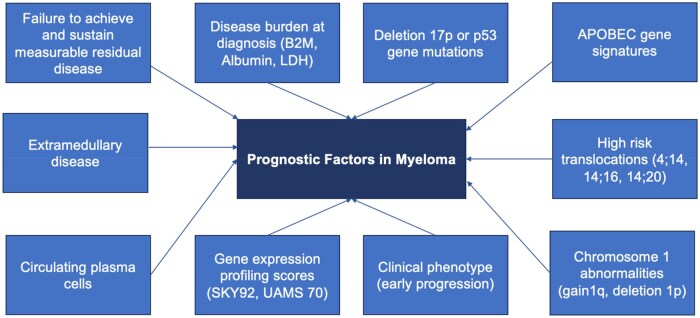
Key prognostic factors in multiple myeloma staging and risk assessment.

Notably, myeloma treatments advance rapidly, and staging systems derived from cohorts of older patients are easily outdated. For example, the standard of care for many patients with myeloma has now shifted from triplets to quadruplets.^67^^,^^68^ Given that the MASS staging system incorporated data from patients treated from 2004 to 2019, and the R2-ISS from patients treated between 2005 and 2016, the patients in these datasets (who were not exposed to anti-CD38 monoclonal antibodies) may not represent patients being treated today with quadruplet therapy and upfront anti-CD38 monoclonal antibodies.^58^^,^^59^ Furthermore, effective staging systems need to strike a balance between being user-friendly and comprehensive. Overly detailed systems might be cumbersome for practical clinical use, while overly simplistic ones might omit critical prognostic factors. Our current staging systems do not account for the genomic complexity of myeloma and its role in prognosis. Key factors of the current staging systems, such as cytogenetic information and beta-2 microglobulin are not always routinely available or obtained in the community setting.^37^ Additionally, other factors that may impart risk in myeloma (Figure 2) are not accounted for in the current staging systems. Key examples of such features are the presence of extramedullary disease and circulating plasma cells.^31^^,^^69–71^

An unsettling occurrence in practice is early progression observed in someone who was otherwise not high-risk at the time of diagnosis, further casting doubt on the validity of current staging systems.^72^ This has led to a coining of the term “functional high-risk,” which has been defined variably in recent literature, but mostly applies this entity to patients progressing within 12-18 months from treatment initiation, despite an optimal initial therapy.^72–74^ This goes to show that dynamic monitoring and risk ascertainment are an integral part of therapy, with early progression necessitating a change in treatment approaches and consideration of novel therapies such as bispecifics and chimeric antigen receptor therapy. Often, these patients are incorrectly classified as having standard-risk disease, due to the failure to isolate CD138 selected cells at the time of diagnosis and perform a cytogenetic analysis on those cells.^38^^,^^75–78^

As quadruplet therapies become the mainstay of induction treatment, previous factors that imparted risk may now be abrogated. In a recent analysis of patients receiving quadruplet therapies,^53^ excellent outcomes were seen in those who had no or a single high-risk cytogenetic abnormality, with poor outcomes confined mostly to those who had 2 or more high-risk cytogenetic abnormalities.^53^ Other studies have also indicated that 30%-40% of individuals with translocation (4; 14), a historic high-risk marker, have results comparable to intermediate- or standard-risk patients.^79^^,^^80^

As our myeloma staging systems have evolved, the role of beta-2 microglobulin ought to be questioned. Beta-2 microglobulin is a component of MHC class I that is secreted from all nucleated cells and cleared by the kidneys.^81^^,^^82^ As a result, kidney dysfunction, regardless of etiology, has a profound impact on beta-2 microglobulin.^83^ Beta-2 microglobulin represents a classic example of tumor burden rather than the underlying biological aggressiveness of the disease, which limits its prognostic accuracy in determining disease outcomes. Although beta-2 microglobulin continues to be carried forward in newer staging systems, its role should be re-evaluated. Furthermore, this has no clinical utility for the care of patients other than for staging purposes and is often not drawn before the start of treatment.^84^

### Beyond traditional staging: other biomarkers

There are other factors that also impact prognosis, such as the tumor microenvironment, which consists of the cells surrounding the tumor, and can play a role in either supporting tumor growth or boosting the immune system’s ability to fight the tumor. In an analysis of 998 bone marrow samples from 436 newly diagnosed patients, a “low-granulocyte” microenvironment was associated with significantly worse progression-free survival and overall survival.^85^ This adverse microenvironment, characterized by low levels of granulocytes, developed as disease progressed and could identify additional high-risk patients beyond those identified by existing clinical criteria. Other work pertaining to the microenvironment includes identification, and preclinical targeting of mesenchymal stem cells that facilitate the proliferation of myeloma cells.^86^

Another factor that impacts prognosis is chromothripsis. Chromothripsis represents chromosomal shattering that occurs in a single catastrophic event, resulting in massive rearrangements within chromosomal regions.^87^ This is present in 20%-30% of newly diagnosed myeloma and is associated with adverse outcomes.^87^

Incorporation of imaging findings, which are not currently utilized in staging systems, can also refine risk assessment. Findings on advanced imaging such as positron emission tomography scan (PET/CT) and diffusion weighted magnetic resonance imaging (MRI) can be prognostic and can help contribute to the refining of staging.^88^^,^^89^ Extra-medullary disease, especially visceral disease (rather than paraosseous disease) is a well-recognized prognostic factor for poor outcomes.^71^ Another example of the prognostic value of imaging is that patients with more than 3 focal lesions on PET/CT have a shortened PFS and OS.^90^ More than 3 focal lesions identified by PET/CT scan or more than 7 lesions identified by axial MRI have been also associated with a high-risk gene expression profile and are more common in extra-medullary disease.^69^ Furthermore, heterogeneity across lesions has been identified through genetic analysis of focal lesions within individual patients, with certain lesions expressing high-risk features and others exhibiting low-risk characteristics.^91^^,^^92^

Another recognized factor that impacts prognosis across the treatment journey is frailty, however this has not yet been incorporated into staging, and routine treatment decision making.^93^^,^^94^

Although staging is historically done at diagnosis, the absence of measurable residual disease (MRD) measured after a period of therapy has emerged as one of the most important prognostic factors in myeloma.^95^ Current trials in myeloma are now using MRD to guide treatment decision making.^96–98^ MRD has already gained approval by the FDA as an intermediate endpoint for drug approval and is being incorporated as such in many ongoing clinical trials.^99^

### The promise of genomic classification systems

Innovative research has revealed mutational genomic aberrations caused by Apolipoprotein B mRNA-editing catalytic polypeptide-like (APOBEC) deaminases, as being critical for myeloma initiation and progression,^100^ and that genomic instability is involved in the pathogenesis of myeloma.^101^ A recent risk prognostication score was derived, incorporating APOBEC signature using data from 1143 patients in the COMPASS dataset, and validating this score in 263 patients treated in a clinical trial.^102^ With a c-index of 0.7 for overall survival, this score outperformed the R-2 ISS (0.67) and R-ISS (0.64).^102^ Although such genomic information is not typically readily available for the average clinician, this study serves as proof of concept for the promise of using genomic data to better predict outcomes. Another example of this is a key recent publication (the IRMMa model), which incorporated genomic factors in prognostication and utilized data from 1993 patients.^103^ This showed superior accuracy that was significantly higher than all other commonly used comparator prognostic models, with a c-index for OS of 0.73, compared with ISS (0.61), revised-ISS (0.57), and R2-ISS (0.63).^103^ The IRMMa model improves risk prediction by integrating APOBEC mutational signatures with structural variants, chromothripsis, and treatment-response data. APOBEC-driven mutagenesis promotes genomic instability which is strongly associated with inferior survival. The IRMMa model could facilitate the individualization of prognosis and treatment selection in myeloma.^103^

Despite these promising advances in genomic classification, significant implementation challenges remain. Genomics will undoubtedly play an increasingly significant role in future staging systems, but to truly transform clinical practice, this information must be both accessible and user-friendly for busy clinicians. This requires an effort to reduce barriers to genomic testing, streamline integration into clinical workflows, and educate healthcare providers. Dedicated randomized controlled trials will be needed in the future, through assessing specific therapies for individual genomic subsets of myeloma and showing that such therapies are better than our current general approach to treating myeloma. A realistic global staging system must balance precision and practicality. While genomics holds promise, cost and infrastructure limit widespread use. Most myeloma patients reside in resource-limited settings throughout the world.

To summarize, the evolution of myeloma staging systems reflects our enhanced understanding of this complex disease. Myeloma staging has evolved from disease burden marker-based systems to those incorporating cytogenetic abnormalities, with a future transition toward genomic profile-based classification systems. Current staging systems reflect dramatic improvements over historical ones yet fail to predict risk for progression in many situations. Individualized approaches to therapy based in high-quality randomized data are needed. Genomics will increasingly play a part in future staging systems, but this must be accessible and easy to use for busy clinicians.

## Data Availability

No new data were generated or analysed in support of this research.

## References

[1] Rees MJ , D’Agostino M, Leypoldt LB, Kumar S, Weisel KC, Gay F. Navigating high-risk and ultrahigh-risk multiple myeloma: challenges and emerging strategies. Am Soc Clin Oncol Educ Book. 2024;44:e433520. 38772002 10.1200/EDBK_433520

[2] Botta C , Ciliberto D, Rossi M, et al. Network meta-analysis of randomized trials in multiple myeloma: efficacy and safety in relapsed/refractory patients. Blood Adv. 2017;1:455-466. 29296961 10.1182/bloodadvances.2016003905 PMC5738982

[3] Caro J , Al Hadidi S, Usmani S, Yee AJ, Raje N, Davies FE. How to treat high-risk myeloma at diagnosis and relapse. Am Soc Clin Oncol Educ Book. 2021;41:291-309. 34010042 10.1200/EDBK_320105

[4] van de Donk NWCJ , Minnema MC, van der Holt B, et al. Treatment of primary plasma cell leukaemia with carfilzomib and lenalidomide-based therapy (EMN12/HOVON-129): final analysis of a non-randomised, multicentre, phase 2 study. Lancet Oncol. 2023;24:1119-1133. 37717583 10.1016/S1470-2045(23)00405-9

[5] Carbone PP , Kellerhouse LE, Gehan EA. Plasmacytic myeloma: a study of the relationship of survival to various clinical manifestations and anomalous protein type in 112 patients. Am J Med. 1967;42:937-948. 6027163 10.1016/0002-9343(67)90074-5

[6] GER Costa , F Taliente, eds. Criteria Defining Risk and Response in Multiple Myeloma. American Association for Cancer Research (AACR); 1969.

[7] Dawson AA , Ogston D. Factors influencing the prognosis in myelomatosis. Postgrad Med J. 1971;47:635-638. 5158835 10.1136/pgmj.47.552.635 PMC2467330

[8] Alexanian R , Balcerzak S, Bonnet JD, et al. Prognostic factors in multiple myeloma. Cancer. 1975;36:1192-1201. 1175123 10.1002/1097-0142(197510)36:4<1192::aid-cncr2820360403>3.0.co;2-i

[9] Durie BGM , Salmon SE. A clinical staging system for multiple myeloma correlation of measured myeloma cell mass with presenting clinical features, response to treatment, and survival. Cancer. 1975;36:842-854. 1182674 10.1002/1097-0142(197509)36:3<842::aid-cncr2820360303>3.0.co;2-u

[10] Prognostic features in the third MRC myelomatosis trial. Medical Research Council’s Working Party on Leukaemia in Adults. Br J Cancer. 1980;42:831-840. 7459218 10.1038/bjc.1980.330 PMC2010574

[11] Merlini G , Waldenström JG, Jayakar SD. A new improved clinical staging system for multiple myeloma based on analysis of 123 treated patients. Blood. 1980;55:1011-1019. 7378577

[12] Cassuto JP , Krebs BP, Viot G, Dujardin P, Masseyeff R. Beta 2 microglobulin, a tumour marker of lymphoproliferative disorders. Lancet. 1978;2:108-109. 10.1016/s0140-6736(78)91428-9 78278

[13] Norfolk D , Child JA, Cooper EH, Kerruish S, Ward AM. Serum beta 2-microglobulin in myelomatosis: potential value in stratification and monitoring. Br J Cancer. 1980;42:510-515. 6159910 10.1038/bjc.1980.273 PMC2010446

[14] Bataille R , Durie BG, Grenier J. Serum beta2 microglobulin and survival duration in multiple myeloma: a simple reliable marker for staging. Br J Haematol. 1983;55:439-447. 6357266 10.1111/j.1365-2141.1983.tb02158.x

[15] Gassmann W , Pralle H, Haferlach T, et al. Staging systems for multiple myeloma: a comparison. Br J Haematol. 1985;59:703-711. 3986136 10.1111/j.1365-2141.1985.tb07366.x

[16] Bataille R , Durie BG, Grenier J, Sany J. Prognostic factors and staging in multiple myeloma: a reappraisal. J Clin Oncol. 1986;4:80-87. 3510284 10.1200/JCO.1986.4.1.80

[17] Durie BG , Stock-Novack D, Salmon SE, et al. Prognostic value of pretreatment serum beta 2 microglobulin in myeloma: a southwest oncology group study. Blood. 1990;75:823-830. 2405920

[18] Greipp PR , San Miguel J, Durie BGM, et al. International staging system for multiple myeloma. J Clin Oncol. 2005;23:3412-3420. 15809451 10.1200/JCO.2005.04.242

[19] Ross FM , Avet-Loiseau H, Ameye G, et al. Report from the European Myeloma Network on interphase FISH in multiple myeloma and related disorders. Haematologica. 2012;97:1272-1277. 22371180 10.3324/haematol.2011.056176 PMC3409827

[20] Fonseca R , Blood E, Rue M, et al. Clinical and biologic implications of recurrent genomic aberrations in myeloma. Blood. 2003;101:4569-4575. 12576322 10.1182/blood-2002-10-3017

[21] Dewald GW , Kyle RA, Hicks GA, Greipp PR. The clinical significance of cytogenetic studies in 100 patients with multiple myeloma, plasma cell leukemia, or amyloidosis. Blood. 1985;66:380-390. 3926026

[22] Fonseca R , Blood EA, Oken MM, et al. Myeloma and the t(11; 14)(q13; q32); evidence for a biologically defined unique subset of patients. Blood. 2002;99:3735-3741. 11986230 10.1182/blood.v99.10.3735

[23] Fonseca R , Harrington D, Oken MM, et al. Biological and prognostic significance of interphase fluorescence in situ hybridization detection of chromosome 13 abnormalities (delta13) in multiple myeloma: an Eastern cooperative oncology group study. Cancer Res. 2002;62:715-720. 11830525

[24] Avet-Loiseau H , Minvielle S, Mellerin MP, Magrangeas F, Bataille R. 14q32 chromosomal translocations: a hallmark of plasma cell dyscrasias? Hematol J. 2000;1:292-294. 11920205 10.1038/sj.thj.6200045

[25] Fonseca R , Bergsagel PL, Drach J, et al. International Myeloma Working Group molecular classification of multiple myeloma: spotlight review. Leukemia. 2009;23:2210-2221. 19798094 10.1038/leu.2009.174 PMC2964268

[26] Dimopoulos MA , Barlogie B, Smith TL, Alexanian R. High serum lactate dehydrogenase level as a marker for drug resistance and short survival in multiple myeloma. Ann Intern Med. 1991;115:931-935. 1952489 10.7326/0003-4819-115-12-931

[27] Terpos E , Katodritou E, Roussou M, et al. High serum lactate dehydrogenase adds prognostic value to the international myeloma staging system even in the era of novel agents. Eur J Haematol. 2010;85:114-119. 20477863 10.1111/j.1600-0609.2010.01466.x

[28] Chng WJ , Dispenzieri A, Chim C-S, et al. IMWG consensus on risk stratification in multiple myeloma. Leukemia. 2014;28:269-277. 23974982 10.1038/leu.2013.247

[29] Palumbo A , Avet-Loiseau H, Oliva S, et al. Revised international staging system for multiple myeloma: a report from International Myeloma Working Group. J Clin Oncol. 2015;33:2863-2869. 26240224 10.1200/JCO.2015.61.2267 PMC4846284

[30] Schavgoulidze A , Perrot A, Cazaubiel T, et al. Prognostic impact of translocation t(14; 16) in multiple myeloma according to the presence of additional genetic lesions. Blood Cancer J. 2023;13:160. 37880285 10.1038/s41408-023-00933-4 PMC10600097

[31] Walker BA , Mavrommatis K, Wardell CP, et al. A high-risk, double-hit, group of newly diagnosed myeloma identified by genomic analysis. Leukemia. 2019;33:159-170. 29967379 10.1038/s41375-018-0196-8 PMC6326953

[32] Shaughnessy J. Amplification and overexpression of CKS1B at chromosome band 1q21 is associated with reduced levels of p27Kip1 and an aggressive clinical course in multiple myeloma. Hematology. 2005;10 Suppl 1:117-126. 16188652 10.1080/10245330512331390140

[33] Fonseca R , Van Wier SA, Chng WJ, et al. Prognostic value of chromosome 1q21 gain by fluorescent in situ hybridization and increase CKS1B expression in myeloma. Leukemia. 2006;20:2034-2040. 17024118 10.1038/sj.leu.2404403

[34] Tokito T , Ushijima M, Hara N, Oda Y, Tsuneyoshi M. Case report 812: well-differentiated osteosarcoma arising in the right third rib. Skeletal Radiol. 1993;22:549-551. 8272897 10.1007/BF00209109

[35] Schmidt TM , Fonseca R, Usmani SZ. Chromosome 1q21 abnormalities in multiple myeloma. Blood Cancer J. 2021;11:83. 33927196 10.1038/s41408-021-00474-8 PMC8085148

[36] Neupane K , Fortuna GG, Dahal R, et al. Alterations in chromosome 1q in multiple myeloma randomized clinical trials: a systematic review. Blood Cancer J. 2024;14:20. 38272897 10.1038/s41408-024-00985-0 PMC10810902

[37] Yu Y , Brown Wade N, Hwang AE, et al. Variability in cytogenetic testing for multiple myeloma: a comprehensive analysis from across the United States. JCO Oncol Pract. 2020;16:e1169-e1180. 32469686 10.1200/JOP.19.00639 PMC7564132

[38] Corre J , Montes L, Martin E, et al. Early relapse after autologous transplant for myeloma is associated with poor survival regardless of cytogenetic risk. Haematologica. 2020;105:e480-e483. 33054068 10.3324/haematol.2019.236588 PMC7556617

[39] Avet-Loiseau H , Attal M, Moreau P, et al. Genetic abnormalities and survival in multiple myeloma: the experience of the intergroupe francophone du myélome. Blood. 2007;109:3489-3495. 17209057 10.1182/blood-2006-08-040410

[40] Thanendrarajan S , Tian E, Qu P, et al. The level of deletion 17p and bi-allelic inactivation of TP53 has a significant impact on clinical outcome in multiple myeloma. Haematologica. 2017;102:e364-e367. 28550191 10.3324/haematol.2017.168872 PMC5685226

[41] Thakurta A , Ortiz M, Blecua P, et al. High subclonal fraction of 17p deletion is associated with poor prognosis in multiple myeloma. Blood. 2019;133:1217-1221. 30692124 10.1182/blood-2018-10-880831 PMC6428662

[42] Shaughnessy JD , Zhan F, Burington BE, et al. A validated gene expression model of high-risk multiple myeloma is defined by deregulated expression of genes mapping to chromosome 1. Blood. 2007;109:2276-2284. 17105813 10.1182/blood-2006-07-038430

[43] Kuiper R , Broyl A, de Knegt Y, et al. A gene expression signature for high-risk multiple myeloma. Leukemia. 2012;26:2406-2413. 22722715 10.1038/leu.2012.127

[44] van Beers EH , van Vliet MH, Kuiper R, et al. Prognostic validation of SKY92 and its combination with ISS in an independent cohort of patients with multiple myeloma. Clin Lymphoma Myeloma Leuk. 2017;17:555-562. 28735890 10.1016/j.clml.2017.06.020

[45] Biran N , Dhakal B, Lentzsch S, et al. Gene expression profiling impacts treatment decision making in newly diagnosed multiple myeloma patients in the prospective PROMMIS trial. EJHaem. 2021;2:375-384. 35844693 10.1002/jha2.209 PMC9175784

[46] van Beers EH , Huigh D, Bosman L, et al. Analytical validation of SKY92 for the identification of high-risk multiple myeloma. J Mol Diagn. 2021;23:120-129. 33152501 10.1016/j.jmoldx.2020.10.010

[47] Kuiper R , Zweegman S, van Duin M, et al. Prognostic and predictive performance of R-ISS with SKY92 in older patients with multiple myeloma: the HOVON-87/NMSG-18 trial. Blood Adv. 2020;4:6298-6309. 33351127 10.1182/bloodadvances.2020002838 PMC7756986

[48] Binder M , Rajkumar SV, Ketterling RP, et al. Prognostic implications of abnormalities of chromosome 13 and the presence of multiple cytogenetic high-risk abnormalities in newly diagnosed multiple myeloma. Blood Cancer J. 2017;7:e600. 28862698 10.1038/bcj.2017.83 PMC5709752

[49] Walker BA , Leone PE, Chiecchio L, et al. A compendium of myeloma-associated chromosomal copy number abnormalities and their prognostic value. Blood. 2010;116:e56-e65. 20616218 10.1182/blood-2010-04-279596

[50] Schavgoulidze A , Cazaubiel T, Perrot A, Avet-Loiseau H, Corre J. Multiple myeloma: heterogeneous in every way. Cancers 2021;13:1285. 33805803 10.3390/cancers13061285 PMC7998947

[51] Lannes R , Samur M, Perrot A, et al. In multiple myeloma, high-risk secondary genetic events observed at relapse are present from diagnosis in tiny, undetectable subclonal populations. J Clin Oncol. 2023;41:1695-1702. 36343306 10.1200/JCO.21.01987 PMC10043564

[52] Sonneveld P , Avet-Loiseau H, Lonial S, et al. Treatment of multiple myeloma with high-risk cytogenetics: a consensus of the International Myeloma Working Group. Blood. 2016;127:2955-2962. 27002115 10.1182/blood-2016-01-631200 PMC4920674

[53] Callander NS , Silbermann R, Kaufman JL, et al. Daratumumab-based quadruplet therapy for transplant-eligible newly diagnosed multiple myeloma with high cytogenetic risk. Blood Cancer J. 2024;14:69. 38649340 10.1038/s41408-024-01030-w PMC11035596

[54] Perrot A , Lauwers-Cances V, Tournay E, et al. Development and validation of a cytogenetic prognostic index predicting survival in multiple myeloma. J Clin Oncol. 2019;37:1657-1665. 31091136 10.1200/JCO.18.00776 PMC6804890

[55] Schmidt TM , Barwick BG, Joseph N, et al. Gain of chromosome 1q is associated with early progression in multiple myeloma patients treated with lenalidomide, bortezomib, and dexamethasone. Blood Cancer J. 2019;9:94. 31767829 10.1038/s41408-019-0254-0 PMC6877577

[56] Weinhold N , Salwender HJ, Cairns DA, et al. Chromosome 1q21 abnormalities refine outcome prediction in patients with multiple myeloma—a meta-analysis of 2,596 trial patients. Haematologica. 2021;106:2754-2758. 34092058 10.3324/haematol.2021.278888 PMC8485656

[57] Shah V , Sherborne AL, Walker BA, et al. Prediction of outcome in newly diagnosed myeloma: a meta-analysis of the molecular profiles of 1905 trial patients. Leukemia. 2018;32:102-110. 28584253 10.1038/leu.2017.179 PMC5590713

[58] D’Agostino M , Cairns DA, Lahuerta JJ, et al. Second revision of the international staging system (R2-ISS) for overall survival in multiple myeloma: a European Myeloma Network (EMN) report within the HARMONY project. J Clin Oncol. 2022;40:3406-3418. 35605179 10.1200/JCO.21.02614

[59] Abdallah NH , Binder M, Rajkumar SV, et al. A simple additive staging system for newly diagnosed multiple myeloma. Blood Cancer J. 2022;12:21. 35102148 10.1038/s41408-022-00611-x PMC8803917

[60] Mohyuddin GR , Rubinstein SM, Kumar S, et al. Performance of newer myeloma staging systems in a contemporary, large patient cohort. Blood Cancer J. 2024;14:95. 38862493 10.1038/s41408-024-01076-w PMC11166956

[61] Boyd KD , Ross FM, Walker BA, et al. Mapping of chromosome 1p deletions in myeloma identifies FAM46C at 1p12 and CDKN2C at 1p32.3 as being genes in regions associated with adverse survival. Clin Cancer Res. 2011;17:7776-7784. 21994415 10.1158/1078-0432.CCR-11-1791 PMC5751883

[62] Avet-Loiseau H , Davies FE, Samur MK, et al. International Myeloma Society/International Myeloma Working Group consensus recommendations on the definition of high-risk multiple myeloma. J Clin Oncol. 2025;43:2739–2751;Jco2401893. 40489728 10.1200/JCO-24-01893 PMC13445576

[63] Derman BA , Kosuri S, Jakubowiak A. Knowing the unknowns in high risk multiple myeloma. Blood Rev. 2022;51:100887. 34479756 10.1016/j.blre.2021.100887

[64] Hartter S , Arand M, Oesch F, Hiemke C. Non-competitive inhibition of clomipramine N-demethylation by fluvoxamine. Psychopharmacology. 1995;117:149-153. 7753960 10.1007/BF02245180

[65] Kaiser MF , Hall A, Walker K, et al. Daratumumab, cyclophosphamide, bortezomib, lenalidomide, and dexamethasone as induction and extended consolidation improves outcome in ultra-high-risk multiple myeloma. J Clin Oncol. 2023;41:3945-3955. 37315268 10.1200/JCO.22.02567

[66] Büttner S , Galjart B, Beumer BR, et al. Quality and performance of validated prognostic models for survival after resection of intrahepatic cholangiocarcinoma: a systematic review and meta-analysis. HPB (Oxford). 2021;23:25-36. 32855047 10.1016/j.hpb.2020.07.007

[67] Sonneveld P , Dimopoulos MA, Boccadoro M, et al. Daratumumab, bortezomib, lenalidomide, and dexamethasone for multiple myeloma. N Engl J Med. 2024;390:301-313. 38084760 10.1056/NEJMoa2312054

[68] Facon T , Dimopoulos M-A, Leleu XP, et al. Isatuximab, bortezomib, lenalidomide, and dexamethasone for multiple myeloma. N Engl J Med. 2024;391:1597-1609. 38832972 10.1056/NEJMoa2400712

[69] Usmani SZ , Heuck C, Mitchell A, et al. Extramedullary disease portends poor prognosis in multiple myeloma and is over-represented in high-risk disease even in the era of novel agents. Haematologica. 2012;97:1761-1767. 22689675 10.3324/haematol.2012.065698 PMC3487453

[70] Fernández de Larrea C , Kyle R, Rosiñol L, et al. Primary plasma cell leukemia: consensus definition by the International Myeloma Working Group according to peripheral blood plasma cell percentage. Blood Cancer J. 2021;11:192. 34857730 10.1038/s41408-021-00587-0 PMC8640034

[71] Bladé J , Beksac M, Caers J, et al. Extramedullary disease in multiple myeloma: a systematic literature review. Blood Cancer J. 2022;12:45. 35314675 10.1038/s41408-022-00643-3 PMC8938478

[72] Gay F , Bertuglia G, Mina R. A rational approach to functional high-risk myeloma. Hematol Am Soc Hematol Educ Program. 2023;2023:433-442. 10.1182/hematology.2023000443 PMC10727111 38066896

[73] Davies FE , Pawlyn C, Usmani SZ, et al. Perspectives on the risk-stratified treatment of multiple myeloma. Blood Cancer Discov. 2022;3:273-284. 35653112 10.1158/2643-3230.BCD-21-0205 PMC9894570

[74] Mateos MV , Martinez BP, Gonzalez-Calle V. High-risk multiple myeloma: how to treat at diagnosis and relapse? Hematol Am Soc Hematol Educ Prog. 2021;2021:30-36. 10.1182/hematology.2021000229 PMC8791132 34889431

[75] Lee H , Duggan P, Chaudhry A, et al. Early relapse for multiple myeloma patients undergoing single autologous stem cell therapy: a single-center experience. Clin Lymphoma Myeloma Leuk. 2018;18:e69-e75. 29158114 10.1016/j.clml.2017.10.009

[76] D’Agostino M , Zaccaria GM, Ziccheddu B, et al. Early relapse risk in patients with newly diagnosed multiple myeloma characterized by next-generation sequencing. Clin Cancer Res. 2020;26:4832-4841. 32616499 10.1158/1078-0432.CCR-20-0951

[77] Kastritis E , Roussou M, Eleutherakis-Papaiakovou E, et al. Early relapse after autologous transplant is associated with very poor survival and identifies an ultra-high-risk group of patients with myeloma. Clin Lymphoma Myeloma Leuk. 2020;20:445-452. 32284296 10.1016/j.clml.2019.10.014

[78] Bygrave C , Pawlyn C, Davies F, et al. Early relapse after high-dose melphalan autologous stem cell transplant predicts inferior survival and is associated with high disease burden and genetically high-risk disease in multiple myeloma. Br J Haematol. 2021;193:551-555. 32524584 10.1111/bjh.16793 PMC11497268

[79] Weinhold N , Heuck CJ, Rosenthal A, et al. Clinical value of molecular subtyping multiple myeloma using gene expression profiling. Leukemia. 2016;30:423-430. 26526987 10.1038/leu.2015.309 PMC4740265

[80] Moreau P , Attal M, Garban F, et al. Heterogeneity of t(4; 14) in multiple myeloma. Long-term follow-up of 100 cases treated with tandem transplantation in IFM99 trials. Leukemia. 2007;21:2020-2024. 17625611 10.1038/sj.leu.2404832

[81] Vincent C , Revillard JP. Beta-2-microglobulin and HLA-related glycoproteins in human urine and serum. Contr Nephrol. 1981;26:66-88. 10.1159/000396105 6169488

[82] Floege J , Bartsch A, Schulze M, Shaldon S, Koch KM, Smeby LC. Clearance and synthesis rates of beta 2-microglobulin in patients undergoing hemodialysis and in normal subjects. J Lab Clin Med. 1991;118:153-165. 1856578

[83] Winchester JF , Salsberg JA, Levin NW. Beta-2 microglobulin in ESRD: an in-depth review. Adv Ren Replace Ther. 2003;10:279-309. 14681859 10.1053/j.arrt.2003.11.003

[84] Jelicic J , Juul-Jensen K, Bukumiric Z, et al. Revisiting beta-2 microglobulin as a prognostic marker in diffuse large B-cell lymphoma. Cancer Med. 2024;13:e7239. 38888359 10.1002/cam4.7239 PMC11184650

[85] Danziger SA , McConnell M, Gockley J, et al. Bone marrow microenvironments that contribute to patient outcomes in newly diagnosed multiple myeloma: a cohort study of patients in the total therapy clinical trials. PLoS Med. 2020;17:e1003323. 33147277 10.1371/journal.pmed.1003323 PMC7641353

[86] Heinemann L , Möllers KM, Ahmed HMM, et al. Inhibiting PI3K-AKT-mTOR signaling in multiple myeloma-associated mesenchymal stem cells impedes the proliferation of multiple myeloma cells. Front Oncol. 2022;12:874325. 35795041 10.3389/fonc.2022.874325 PMC9251191

[87] Maclachlan KH , Rustad EH, Derkach A, et al. Copy number signatures predict chromothripsis and clinical outcomes in newly diagnosed multiple myeloma. Nat Commun. 2021;12:5172. 34453055 10.1038/s41467-021-25469-8 PMC8397708

[88] Moulopoulos LA , Gika D, Anagnostopoulos A, et al. Prognostic significance of magnetic resonance imaging of bone marrow in previously untreated patients with multiple myeloma. Ann Oncol. 2005;16:1824-1828. 16087694 10.1093/annonc/mdi362

[89] Kraeber-Bodéré F , Zweegman S, Perrot A, et al. Prognostic value of positron emission tomography/computed tomography in transplant-eligible newly diagnosed multiple myeloma patients from CASSIOPEIA: the CASSIOPET study. Haematologica. 2023;108:621-626. 36263839 10.3324/haematol.2021.280051 PMC9890028

[90] Usmani SZ , Mitchell A, Waheed S, et al. Prognostic implications of serial 18-fluoro-deoxyglucose emission tomography in multiple myeloma treated with total therapy 3. Blood. 2013;121:1819-1823. 23305732 10.1182/blood-2012-08-451690 PMC3591801

[91] Rasche L , Chavan SS, Stephens OW, et al. Spatial genomic heterogeneity in multiple myeloma revealed by multi-region sequencing. Nat Commun. 2017;8:268. 28814763 10.1038/s41467-017-00296-y PMC5559527

[92] Rasche L , Angtuaco EJ, Alpe TL, et al. The presence of large focal lesions is a strong independent prognostic factor in multiple myeloma. Blood. 2018;132:59-66. 29784643 10.1182/blood-2018-04-842880 PMC6034645

[93] Groen K , Smits F, Nasserinejad K, et al. Assessing frailty in myeloma: the pursuit of simplicity may sacrifice precision of predicting clinical outcomes. Hemasphere. 2024;8:e85. 38966208 10.1002/hem3.85 PMC11223651

[94] Mian H , McCurdy A, Giri S, et al. The prevalence and outcomes of frail older adults in clinical trials in multiple myeloma: a systematic review. Blood Cancer J. 2023;13:6. 36599867 10.1038/s41408-022-00779-2 PMC9813365

[95] Munshi NC , Avet-Loiseau H, Rawstron AC, et al. Association of minimal residual disease with superior survival outcomes in patients with multiple myeloma: a meta-analysis. JAMA Oncol. 2017;3:28-35. 27632282 10.1001/jamaoncol.2016.3160 PMC5943640

[96] Derman BA , Major A, Cooperrider J, et al. Discontinuation of maintenance therapy in multiple myeloma guided by multimodal measurable residual disease negativity (MRD2STOP). Blood Cancer J. 2024;14:170. 39375362 10.1038/s41408-024-01156-x PMC11458825

[97] Rosiñol L , Oriol A, Ríos R, et al. Lenalidomide and dexamethasone maintenance with or without ixazomib, tailored by residual disease status in myeloma. Blood. 2023;142:1518-1528. 37506339 10.1182/blood.2022019531

[98] Krishnan A , Hoering A, Hari P, Sexton R, Orlowski RZ. Phase III. Study of daratumumab/rhuph20 (nsc- 810307) + lenalidomide or lenalidomide as post-autologous stem cell transplant maintenance therapyin patients with multiple myeloma (mm) using minimal residual disease to direct therapy duration (DRAMMATIC study): SWOG s1803. Blood. 2020;136:21-22.

[99] Landgren O , Prior TJ, Masterson T, et al. EVIDENCE meta-analysis: evaluating minimal residual disease as an intermediate clinical end point for multiple myeloma. Blood. 2024;144:359-367. 38768337 10.1182/blood.2024024371 PMC11418064

[100] Walker BA , Wardell CP, Murison A, et al. APOBEC family mutational signatures are associated with poor prognosis translocations in multiple myeloma. Nat Commun. 2015;6:6997. 25904160 10.1038/ncomms7997 PMC4568299

[101] Neuse CJ , Lomas OC, Schliemann C, et al. Genome instability in multiple myeloma. Leukemia. 2020;34:2887-2897. 32651540 10.1038/s41375-020-0921-y

[102] Grasedieck S , Panahi A, Jarvis MC, et al. Redefining high risk multiple myeloma with an APOBEC/inflammation-based classifier. Leukemia. 2024;38:1172-1177. 38461190 10.1038/s41375-024-02210-0 PMC11073955

[103] Maura F , Rajanna AR, Ziccheddu B, et al. Genomic classification and individualized prognosis in multiple myeloma. J Clin Oncol. 2024;42:1229-1240. 38194610 10.1200/JCO.23.01277 PMC11095887

